# Raymond Levy PhD, FRCP (E), FRCPsych

**DOI:** 10.1192/bjb.2025.15

**Published:** 2025-10

**Authors:** Robert Howard, Robin Jacoby

Formerly Professor of Old Age Psychiatry, Institute of Psychiatry and Consultant Psychiatrist, Maudsley Hospital, London

**Figure:**
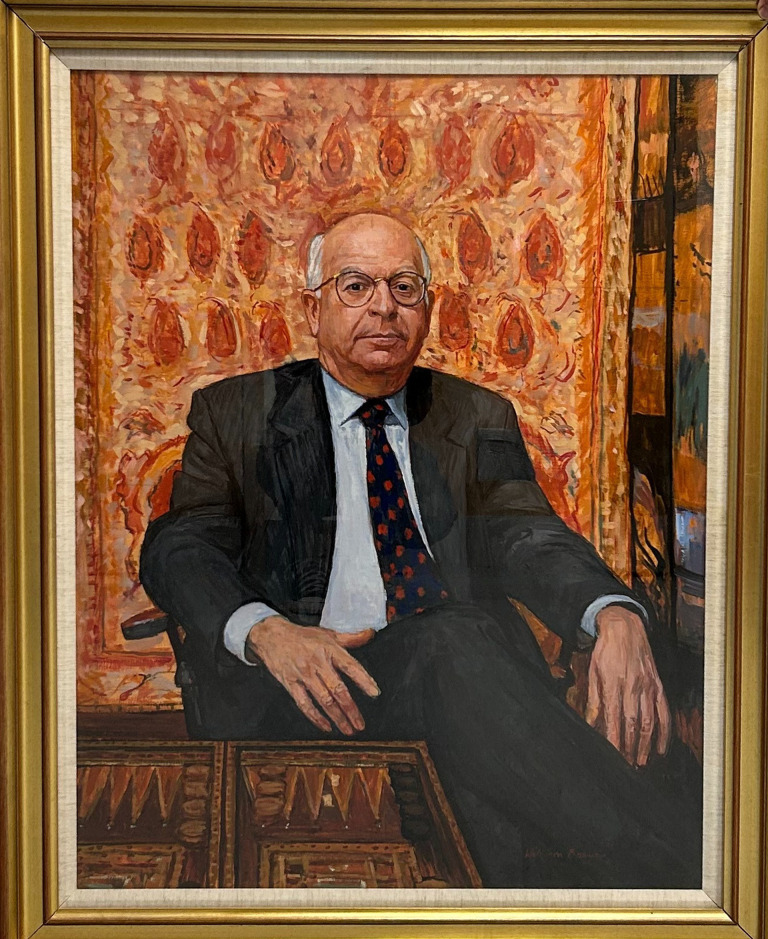

Raymond Levy, who died on 29 September 2024, aged 91, was an international leader of biological research in older people's mental health and dementia, and his original studies of auditory-evoked potentials in dementia and depression, computed tomography of the brain in dementia and depression and clinical trials of the acetylcholine precursor lecithin and the cholinesterase inhibitor tacrine set the scene for much of the research in the field over the last 40 years. In particular, his single-centre tacrine effectiveness trial demonstrated the benefits and limitations of cholinesterase inhibitor therapy and, were it not for the hepatotoxicity of the drug, this would have enjoyed massive success as the first effective symptomatic treatment for Alzheimer's disease. He established and led the Institute of Psychiatry memory clinic, an early prototype of the dementia diagnosis clinics that are now part of every National Health Service clinical service, and which, in the 1980s, served as a feed of carefully characterised and motivated participants for dementia treatment trials.

Generous and long-sighted in his approach to younger colleagues, Raymond involved himself wholeheartedly in the support of the next generation of academic old age psychiatrists. He had a gift for spotting research potential and, once he had involved you in an area of scientific inquiry, would prove a forthright and steadfast supporter. Often as interested in people as he was in the scientific work they were pursuing, he openly enjoyed the subsequent success of those who had trained with him as they proceeded to chairs in old age psychiatry (Osvaldo Almeida, David Ames, Alistair Burns, Engin Ecker, Hans Forstl, Clive Holmes, Robert Howard, Robin Jacoby, Simon Lovestone and John O'Brien), other areas of psychiatry (Melanie Abas and Declan McLachlan) or psychology (Adrian Owen and Barbara Sahakian). Somehow, the heady mixture of cigar smoke, anecdote and penetrating and usually positive observation that constituted supervision was highly effective, and we all thrived in his care. The generation that he supported and promoted has undoubtedly applied much of what we experienced from Raymond, but none of us have matched him in terms of successful supervisees.

Raymond Levy was born on 21 June 1933 into a French-speaking Sephardi Jewish family in Cairo. His father was Gaston Levy, a businessman, and his mother, Esther, a housewife. Educated at Cairo's Victoria College, he was a contemporary and school friend of Omar Sharif and liked to recall that they appeared together in school plays. He came to Edinburgh for undergraduate medical studies, training in neurology, and gained the MRCP (E), after which he obtained a doctorate in neurophysiology. He then moved to the Maudsley Hospital to train as a psychiatrist, arriving in the heyday of Aubrey Lewis’ leadership. After a senior lecturer appointment at the Middlesex Hospital, where, with Vic Meyer, he pioneered the behavioural treatment of obsessive-compulsive disorder, he returned to the Maudsley, where Felix Post had been conducting seminal research into the treatment of mental disorders in older people. Most psychiatrists got to know Raymond when he was Professor of Old Age Psychiatry at the Institute of Psychiatry and actively involved in clinical work through the Felix Post Unit Day Hospital at the Maudsley. His intelligence, sociability, cosmopolitan background and humour made Raymond a popular colleague, trainer and a reliably entertaining canteen companion.

Raymond retired in 1996 and after serving as President of the International Psychogeriatric Association devoted his time to tennis, scuba-diving, art, travel and writing a book on his native Cairo.

Divorced from his first wife Katherine in 1979, Raymond is survived by his daughters, Simone and Tanya, and two grandchildren. He married the psychiatrist Aykan Pulular in 2012 after they had met at a conference in Istanbul. They were a devoted couple and, as Raymond grew older, Aykan looked after him tenderly and with great devotion.

